# Mitochondrial haplogroup G is associated with nonalcoholic fatty liver disease, while haplogroup A mitigates the effects of PNPLA3

**DOI:** 10.1002/edm2.187

**Published:** 2020-10-06

**Authors:** Aaron M. Gusdon, You Hui, Jing Chen, Clayton E. Mathews, Shen Qu

**Affiliations:** ^1^ Department of Neurosurgery Mischer Neuroscience Associates University of Texas Health Science Center at Houston Houston TX USA; ^2^ Department of Endocrinology Shanghai Tenth People's Hospital Tongji University Shanghai China; ^3^ Department of Pathology, Immunology and Laboratory Medicine University of Florida College of Medicine Gainesville FL USA

**Keywords:** haplogroups, mitochondria, NAFLD, PNPLA3

## Abstract

**Objectives:**

Mitochondrial dysfunction plays a pivotal role in the pathogenesis of nonalcoholic fatty liver disease (NAFLD). We hypothesized that mitochondrial DNA (mtDNA) haplogroups affect the risk of NAFLD in Han Chinese patients and interact with PNPLA3 genotypes.

**Design:**

NAFLD and control patients were recruited from a tertiary care centre. The mitochondrial genome was amplified in overlapping segments and sequenced. Mitochondrial haplogroups were determined using Mitomaster. *PNPLA3* rs738409 genotyping was performed using restriction fragment length polymorphism analysis.

**Patients:**

We enrolled 655 NAFLD patients and 504 controls.

**Results:**

More NAFLD patients encoded haplogroup G; odds ratio (OR) 1.85 (95% confidence interval [CI] 1.16, 2.80). Subhaplogroup G3 was present more frequently in NAFLD patients (25.8% vs 6.5%). The *PNPLA3 *CG genotype resulted in an OR of 1.66 (95% CI 1.25, 2.21), and the GG genotype resulted in an OR of 2.33 (95% CI 1.72, 3.17) for NAFLD. Patients with mitochondrial haplogroup A had a significantly higher frequency of genotype GG. Among patients with haplogroup A, no *PNPLA3* genotype was associated with increased NAFLD risk (CG: OR 1.17, 95% CI 0.55, 2.34; GG: OR 1.04 95% CI 0.66, 2.65). Excluding haplogroup A, the OR for CG was 1.58 (95% CI 1.18, 2.12), and the OR for GG was 1.81 (95% CI 1.30, 2.51).

**Conclusion:**

Haplogroup G was associated with an increased risk of NAFLD *PNPLA3 *GG genotype was overrepresented among patients encoding haplogroup A and was not associated with NAFLD risk among haplogroup A patients. Mitochondrial genetics influence NAFLD risk and interact with *PNPLA3* genotypes.

## Graphical abstract

Mitochondrial genetics affects NAFLD. Mitochondrial haplogroup G is associated with a higher incidence of NAFLD. Mitochondrial haplogroup A mitigates the pathogenicity of the PNPLA32 GG allele.

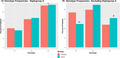

## 1 INTRODUCTION

Nonalcoholic fatty liver disease (NAFLD) encompasses a spectrum of disorders beginning with hepatic steatosis, which can progress to steatohepatitis, cirrhosis and hepatocellular carcinoma in a subset of patients.^1^ The prevalence of NAFLD has risen worldwide along with increases in type 2 diabetes, obesity and metabolic syndrome and is estimated at approximately 25%.^2^ The prevalence of NAFLD in East Asian is particularly high (estimated at 27%).^2^

Mitochondria play an important role in the pathogenesis of NAFLD. After an initial induction of mitochondrial function, an increased amount of liver fat leads to systemic changes in metabolism and reduced metabolic flexibility.^3^ Liver mitochondrial fatty acid oxidation (FAO) plays an important role in metabolizing the influx of hepatic fatty acids in NAFLD. The increased flux of metabolites through the electron transport chain (ETC) results in production of reactive oxygen species (ROS) and subsequent oxidative stress.^4^ ROS and oxidative stress can disrupt function of the ETC, alter mitochondrial membrane potential and trigger mitochondrial permeability transition pore formation.^5^ Indeed, these patients exhibit enlarged and swollen mitochondria and have impairment in the enzymatic activity of the ETC.^6^, ^7^

In additional to affecting enzymatic function, ROS can also damage mitochondrial DNA (mtDNA). NAFLD patients have been shown to have decreased mtDNA copy numbers and epigenetic modifications impairing expression of genes encoding ETC components.^8^ Furthermore, liver mtDNA in patients with NAFLD has been shown to have a higher mutational rate and degree of heteroplasmy.^8^ Importantly, defining groups of mtDNA polymorphisms termed haplogroups have been shown to influence susceptibility to a variety of disorders.^9^ In one population, haplogroup L was found to be protective against the development of steatohepatitis.^10^

Nuclear genes associated with NAFLD have been extensively investigated, with an isoleucine to methionine substitution in *PNPLA3* being the most robust variant linked to the development of NAFLD.^11^ *PNPLA3* encodes a protein with hydrolase activity acting on triglycerides and retinyl esters. Loss of function results in accumulation of triglycerides and retinyl esters in lipid droplets of hepatocytes and hepatic stellate cells.^12^ Complex interactions between genetic and environmental factors contribute to the development and progression of NAFLD. The nuclear and mitochondrial genomes are intricately associated and have co‐evolved.^13^ Interactions between the nuclear and mitochondrial genomes have been demonstrated in several disease processes^14^, ^15^, ^16^, ^17^ and have also been shown to modulate whole body metabolism.^18^ Nuclear and mitochondrial genomes are known to interact in NAFLD, with varying degrees of liver steatosis and mitochondrial function on different nuclear DNA backgrounds.^14^ Importantly, the mitochondrial genome differs considerably comparing Eastern and Western populations, and mtDNA is known to modulate the nuclear genome.^19^

Mitochondria play a key role in the pathogenesis of NAFLD.^5^, ^8^, ^20^ Mitochondrial haplogroups affect mitochondrial function and have co‐evolved with the nuclear genome.^13^, ^17^ We therefore hypothesized that East Asian mitochondrial DNA haplogroups will play a role in the development of NAFLD and affect the penetrance of *PNPLA3* polymorphisms.

## 2 MATERIAL AND METHODS

### 2.1 Patient selection

Patients were enrolled from a tertiary medical centre, Shanghai Tenth People's Hospital affiliated with Tongji University School of Medicine. Informed consent was obtained from each patient. This study conformed to the ethical guidelines of the 1975 Declaration of Helsinki and was approved by the institutional review board of Shanghai Tenth People's Hospital. Patients with NAFLD were recruited from the endocrinology clinic. In order to be included, NAFLD patients must have had no history of drinking or for men drink <140 g and for women drink <70 g of alcohol per week. Grams of alcohol was calculated as alcohol consumption (mL) × alcohol content (%) × 0.8 (alcohol‐specific gravity). NAFLD patients also must have had no history of other liver‐related diseases including hepatitis A, B and D or liver cirrhosis. Serology was performed for each patient in order to rule out viral hepatitis. All NAFLD patients also had ultrasound findings suggestive of fatty liver disease. Qualitative measures of were assessed including echogenicity, hepatomegaly and intrahepatic vascular blurring as described.^21^ Quantitative ultrasonography was performed for each patient to evaluate fat content and fibrosis. Liver fat quantification was performed using the back‐scatter coefficient, which measures the returned ultrasound energy from tissues.^22^ This method has been shown to be more accurate than conventional ultrasonography.^23^ To be included in the current study, patients had to have ≥ grade 1 steatosis. Transient elastography was used to measure hepatic elasticity as described,^24^, ^25^, ^26^ with lower values used to exclude advanced fibrosis.^27^ Control patients were recruited during routine physical examination. To be included as a control, no history of NAFLD, type 2 diabetes mellitus (T2DM) or type 1 diabetes mellitus (T1DM) could be present based on medical history and laboratory results including blood glucose and haemoglobin A1c. NAFLD was excluded by negative liver ultrasound results. Control patients also had no history of viral hepatitis or cirrhosis based on medical history, serology and liver ultrasound results.

Demographic and laboratory data were collected for each patient. Weight and height were determined using Omron HBF‐358, with each patient only wearing light clothing without shoes or hats. Body mass index (BMI) was calculated as weight in kilograms divided by height in metres squared. Morning venous blood was obtained from all patients after a 12‐hour fast, with each sample then centrifuged and the supernatant used for laboratory testing. Alanine aminotransferase (ALT), aspartate aminotransferase (AST), gamma‐glutamyl transferase (GGT), triglyceride (TG), total cholesterol (TC), high‐density lipoprotein cholesterol (HDL‐C), low‐density lipoprotein cholesterol (LDL‐C), total protein (TP), albumin, alkaline phosphatase (ALP), direct bilirubin(DBili) and total bilirubin (TBili) were assessed using an automated biochemical analyser.

### 2.2 Mitochondrial haplogroup determination

The mitochondrial genome was amplified in four segments. Sanger sequencing was performed to obtain the complete mitochondrial genome. Mitochondrial DNA sequences were converted to FASTA files. Mitochondrial DNA haplogroups were determined using Mitomaster (https://www.mitomap.org/mitomaster/index.cgi) as described.^28^

### 2.3 *PNPLA3* genotyping

The *PNPLA3* rs738409 polymorphism was genotyped as described using restriction fragment length polymorphism (RFLP) analysis.^29^ Initially, the region of interest was amplified with conventional PCR (forward primer 5′‐TGGGCCTGAAGTCCGAGGGT‐3′, reverse primer 5′‐CCGACACCAGTGCCCTGCAG‐3′). A PCR product of 333 base pairs (bp) was amplified. Following amplification, PCR products were digested with the restriction enzyme BtsCI (New England Biolabs). Patients who were CC homozygous demonstrated 200 and 133 bp restriction fragments. Patients who were CG heterozygous demonstrated 333, 200 and 133 bp fragments. Patients who were homozygous for the GG mutant allele demonstrated a single 333 bp fragment. An example RFLP result is shown in Figure S1.

### 2.4 Statistical methods

All statistical analysis was performed using R (R Project for Statistical Computing). Comparisons between each demographic variable were conducted using a Mann‐Whitney *U* test. Comparisons between mitochondrial haplogroup frequencies were performed using Fisher's exact tests as described.^30^ Odds ratios and confidence intervals were calculated using the Functions for Medical Statistics Book (‘fmsb’) package in R *P‐*values were corrected for multiple comparisons using Bonferroni corrections.

## 3 RESULTS

### 3.1 Demographics

A total of 655 patients with NAFLD and 504 control patients were included. Patient demographics are shown in Table 1. Age and gender were not significantly different between control and NAFLD patients. NAFLD patients weighed significantly more and had higher BMI. Triglycerides were higher, and HDL was lower in patients with NAFLD, while there was no significant difference in cholesterol or LDL. NAFLD patients had higher AST, GGT and ALP compared with control, while ALT was not significantly different. Albumin and total protein levels were similar between control and NAFLD patients.

**TABLE 1:** Demographic characteristics of each group

	Control	NAFLD	*P‐*value
N	504	655	
Age	34 (28, 43)	39 (31, 52)	.06
Female gender	202 (41.5)	288 (44.0)	.720
BMI (kg/m^2^)	22.0 (20.2, 23.9)	26.3 (24.4, 28.4)	1.4 × 10^−8^
Fasting blood glucose (mmol/L)	4.7 (4.5, 5.0)	4.9 (4.6, 5.3)	.701
Triglycerides (mmol/L)	0.96 (0.84, 1.14)	1.99 (1.51, 2.70)	.007
Cholesterol (mmol/L)	5.11 (4.12, 5.71)	4.97 (4.29, 5.45)	.977
HDL (mmol/L)	1.43 (1.26, 1.69)	1.09 (0.95, 1.30)	.011
LDL (mmol/L)	2.95 (2.29, 3.48)	3.25 (2.59, 3.79)	.655
ALT (U/L)	16.4 (11.3, 32.5)	22.9 (20.4, 30.95)	.394
AST (U/L)	17.5 (15.0, 20.9)	21.0 (18.1, 26.0)	.023
GGT (U/L)	18.6 (15.8, 32.1)	44.0 (28.5, 50.5)	.008
ALP (U/L)	66.7 (57.2, 80.3)	79.6 (67.6, 89.9)	4.18 × 10^−6^
Total Bilirubin (μmol/L)	12.8 (9.4, 17.5)	12.0 (8.6, 15.6)	.360
Direct Bilirubin (μmol/L)	3.70 (2.78, 4.60)	3.15 (2.40, 4.1)	.07
Albumin (g/L)	49.0 (48.1, 51.2)	50.1 (48.2, 51.3)	.437
Total protein (g/L)	75.3 (72.1, 78.5)	77.1 (73.4, 79.8)	.291

Abbreviations: ALP, alkaline phosphatase; ALT, alanine aminotransferase; AST, aspartate aminotransferase; BMI, body mass index; GGT, gamma‐glutamyl transferase; HDL, high‐density lipoprotein; LDL, low‐density lipoprotein.

### 3.2 Mitochondrial haplogroup frequencies

Frequencies of each macrohaplogroup were determined for both control and NAFLD patients. A significantly higher percentage of patients with NAFLD encoded haplogroup G compared with controls (Table 2). The odds ratio (OR) for NAFLD for haplogroup G was 1.85 (95% confidence interval [95% CI] 1.16, 2.80). The effect of haplogroup G was present after correcting for confounding variables including sex in multivariate models (Table 3). Among those with haplogroup G, subhaplogroup G3 was represented more frequently in patients with NAFLD (N = 16, 25.8%) vs control (N = 4, 6.5%). Of note, there was no significant difference between the percentage of control and NAFLD patients encoding mitochondrial haplogroup D (Table 2). Since haplogroup D and some of its subhaplogroups have been implicated as being protective against a variety of age‐related disorders,^31^, ^32^ we also looked at the frequency of each of the D subhaplogroups. Among those individuals encoding haplogroup D, there were no significant differences between the percentage of control and NAFLD patients considering each subhaplogroup (Table S1).

**TABLE 2:** Frequencies of each mitochondrial haplogroup

	A	B	C	D	E	F	G	H	K	L	M	N	R	T	U	Y	Z
Control	41^a^ (8.1)	57 (11.3)	17 (3.4)	159 (24.2)	1 (0.2)	68 (13.5)	17 (3.4)	29 (5.8)	7 (1.1)	2 (0.3)	90 (17.9)	21 (4.2)	8 (1.6)	2 (0.3)	3 (0.5)	4 (0.8)	15 (3.0)
NAFLD	44 (6.7)	80 (12.2)	31 (4.7)	127 (25.2)	1 (0.2)	74 (11.3)	64 (9.8)	36 (5.5)	6 (1.2)	1 (0.2)	121 (18.5)	22 (3.4)	14 (2.1)	1 (0.2)	3 (0.6)	8 (1.2)	16 (2.4)
*P*‐value	.91	.69	.25	.37	.88	.81	**.01**	.77	.41	.52	.39	.84	.26	.52	.68	.19	.76
OR	0.82	1.09	1.45	1.01	0.37	0.82	1.85	0.79	1.30	1.48	1.05	0.77	1.74	1.48	0.74	1.98	0.78
95% CI	0.53, 1.29	0.75, 1.56	0.79, 2.69	0.76, 1.32	0.03, 4.08	0.58, 1.17	1.16, 2.80	0.49, 1.29	0.38, 4.45	0.13, 16.4	0.77, 1.42	0.42, 1.41	0.66, 4.56	0.13, 16.4	0.15, 3.67	0.52, 7.51	0.38, 1.60

^a^ Results are presented as N (%).

Bold values denote statistical significance.

**TABLE 3:** Multivariate regression models for prevalence of NAFLD considering Haplogroup G

	Model 1	Model 2	Model 3
Haplogroup G	1.84 (1.14, 2.78)^a^	1.78 (1.12, 2.67)	1.51 (1.06, 2.77)
Covariates			
BMI (kg/m^2^)	1.54 (1.46, 1.62)	1.53 (1.46, 1.62)	1.52 (1.45, 1.63)
Triglycerides (mmol/L)	1.22 (1.12, 1.32)	1.23 (1.13, 1.32)	1.20 (1.10, 1.31)
HDL (mmol/L)	0.971 (0.952, 0.992)	0.980 (0.965, 0.994)	0.981 (0.966, 0.996)
AST (U/L)		1.02 (1.01, 1.03)	1.03 (1.01, 1.04)
GGT (U/L)		1.017 (0.997, 1.036)	1.016 (0.998, 1.037)
ALP (U/L)		1.002 (0.999, 1.005)	1.001 (0.998, 1.004)
Fasting Blood Glucose (mmol/L)			1.023 (0.996, 1.051)
Female sex			0.820 (0.710, 0.931)
Female sex and age >50			0.998 (0.623, 1.38)

Abbreviations: ALP, alkaline phosphatase; AST, aspartate aminotransferase; BMI, body mass index; GGT, gamma‐glutamyl transferase; HDL, high‐density lipoprotein; OR, odds ratio.

^a^ OR (95% CI).

### 3.3 Association with PNPLA3 genotypes

*PNPLA3* genotypes were determined for each patient, and the frequencies of homozygous wild type (CC), heterozygous (CG) and homozygous mutant (GG) were calculated. Among control patients, the percentage of CC, CG and GG genotypes were 37.6, 37.8 and 24.6, respectively. Among NAFLD patients, the percentage of CC, CG and GG genotypes were 23.8, 39.8 and 36.4, respectively. Among heterozygous patients (CG), the OR for NAFLD was 1.66 (95% CI 1.25, 2.21; *P = *4.57 × 10^−4^. Among homozygous patients (GG), the OR for NAFLD was 2.33 (95% CI 1.72, 3.17; *P* = 5.01 × 10^−8^).

**FIGURE 1:**
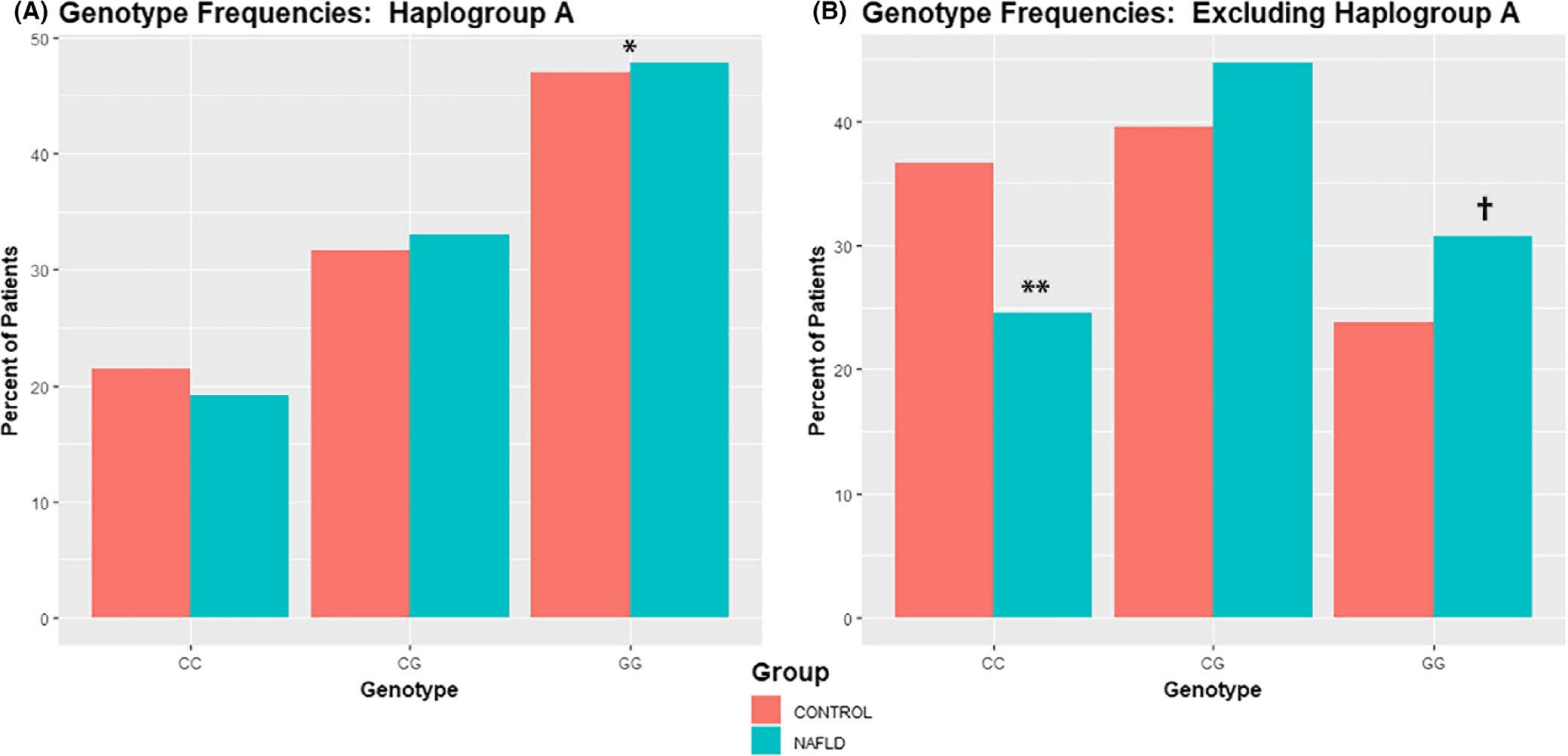
Frequency of PNPLA3 rs738409 genotypes. The frequency of each *PNPLA3* genotype is shown considering only those patients with haplogroup A (A). The frequency of each *PNPLA3* genotype is shown considering patients with all haplogroups except A (B). *GG vs CC and CG, *P* < .05; **Control vs NAFLD, *P* < .05; ^†^Control vs NAFLD, *P* < .05

Patients were further categorized according to mitochondrial haplogroup. Among patients with haplogroup A, the frequency of the GG genotype was significantly higher than both CC and CG (Table S2). There was no difference in the frequencies of each *PNPLA3* genotype among the other haplogroups. When considering only patients with haplogroup A (Figure 1A), a significantly higher percentage of control and NAFLD patients encoded the GG haplotype. However, there was no difference in the frequency of each allele comparing control and NAFLD patients. Among patients with haplogroup A who were heterozygous (CG), the OR for NAFLD was 1.17 (95% CI 0.55, 2.34; *P* = .65). Among patients with haplogroup A who were homozygous (GG), the OR for NAFLD was 1.04 (95% CI 0.66, 2.65; *P = *.69). Among all patients excluding those with haplogroup A (Figure 1B), a higher frequency of patients with NAFLD encoded the CG and GG alleles, while fewer encoded the wild‐type CC allele. Among all patients excluding haplogroup A who were heterozygous (CG), the OR for NAFLD was 1.58 (95% CI 1.18, 2.12; *P = *.002). Among all patients excluding haplogroup A who were homozygous (GG), the OR for NAFLD was 1.81 (95% CI 1.30, 2.51, *P* = 3.76 × 10^−4^). These associations remained significant in multivariate regressions models (Table 4).

**TABLE 4:** Multivariate regression models for prevalence of NAFLD according to *PNPLA3* genotype

	Haplogroup A (N = 97)	All excluding haplogroup A (N = 1062)
*PNPLA3* GG	1.02 (0.63, 2.61)^a^	1.74 (1.26, 2.48)
Covariates		
BMI (kg/m^2^)	1.32 (1.28, 1.38)	1.50 (1.42, 1.60)
Triglycerides (mmol/L)	1.15 (1.09, 1.24)	1.22 (1.12, 1.30)
HDL (mmol/L)	0.985 (0.980, 0.991)	0.979 (0.961, 0.992)
AST (U/L)	1.02 (0.89, 1.09)	1.03 (1.02, 1.04)
Female sex	0.823 (0.519, 1.13)	0.872 (0.782, 0.964)
Female sex and age >50	0.992 (0.732, 1.25)	0.998 (0.819, 1.18)

Abbreviations: AST, aspartate aminotransferase; BMI, body mass index; HDL, high‐density lipoprotein; OR, odds ratio.

^a^ OR (95% CI).

## 4 DISCUSSION

In this study, we sought to determine whether mtDNA haplogroups affected the development of NAFLD and modulated the effects of the *PNPLA3* rs738409 polymorphism. We found that haplogroup G was significantly more common in NAFLD patients compared with controls. We also found that among patients with haplogroup A, there was a higher frequency of the pathogenic rs738409 GG genotype.

Variations in mtDNA can be adaptive or maladaptive, with variations clustering together along discrete branches of the human mtDNA tree to form haplogroups.^9^ Each haplogroup is defined by one or more functional variations,however, a number of variations have arisen across different populations, indicating a potential adaptive selection.^33^ Haplogroups also correlate with geographic distribution of populations. Haplogroup L is found in sub‐Saharan Africa and gave rise to haplogroups M and N. Only haplogroup N moved north into Europe, while both M and N migrated towards Asia with M giving rise to the Asian haplogroups C, D and G, among others, and N giving rise to haplogroups such as A, B and F.^17^ In addition to defining patterns of human migration, haplogroups have been shown to affect clinical conditions. Haplogroup J was shown to modulate the penetrance of Leber's hereditary optic neuropathy (LHON) mutations.^34^ Haplogroup J correlates with increased longevity in Europeans,^35^ while haplogroup D is correlated with increased longevity in Japanese.^36^ Of note, haplogroup D has also been associated with decreased risk of type 1 diabetes.^31^ Other haplogroups have also been shown to modulate the risk of diabetes with N9a being protective against diabetes and metabolic syndrome in Asians^37^ and J being associated with an increased risk of diabetes in European populations.^38^ Haplogroup defining mutations often result in functional changes in the ETC, and it has been suggested that these mutations are adaptive to certain climates.^17^ Macrohaplogroup N acquired functional variants in *ND3* and *ATP6*, which result in alterations in membrane potential and coupling efficiency.^39^ The *ND1* T3394C polymorphism decreases complex I activity and increases penetrance of LHON mutations when present in the context of macrohaplogroup N, while it may be beneficial to certain high altitude populations in the context of haplogroup M9.^15^

Haplogroup G is an Asian haplogroup, the phylogeny of which has only relatively recently been thoroughly characterized and refined.^40^ Haplogroup G is defined by a 4833 transition and 16 362, 5108 and 709 mutations. The G3 subhaplogroup is defined by a 16 274 transition,^41^ with G3a defined by mutations at 143 and 15 746 and G3b defined by mutations at 13 477, 14 605 and 15 927. Relatively little research has focused on the role of haplogroup G. A recent study demonstrated that haplogroup G is associated with an increased prevalence of metabolic syndrome in Han Chinese patients.^42^ Another recent study found an association with haplogroup G and osteoarthritis.^43^ In 2000, without noting it as the defining feature of haplogroup G, Ohkubo et al^44^ reported that the A4833G transition is associated with impaired glucose tolerance. This mutation results in an amino acid change from threonine to alanine (T122A) in the ND2 submit of NADH dehydrogenase (mitochondrial complex I). In cybrid cells, this mutation was shown to decrease complex I activity by 35% without affecting the function of complex IV.^44^ It is therefore tempting to speculate that decreased complex I activity due to the haplogroup G A4833G mutation may contribute to susceptibility to NAFLD. It is conceivable that decreased complex I activity results in slower fatty acid oxidation and therefore a propensity to accumulate liver fatty acids. However, much is unknown about how A4833G affects mitochondrial function, for instance whether it affects ROS production or membrane potential and coupling, both of which could have significant effects on fatty acid metabolism and the development of NAFLD.

Of note, we did not find a significant association between haplogroup D or any of its subhaplogroups and NAFLD. Haplogroup D has been associated with various age‐related disorders^45^ and has been shown to modulated ROS production.^31^ Importantly, haplogroup D has not been demonstrated to affect the enzymatic activity of complex I or coupling of the ETC.^46^ Therefore, it likely does not affect fatty acid metabolism. Haplogroup D may therefore not be expected to affect the accumulation of hepatic fatty acids. However, our study was not designed to assess progression from steatosis to steatohepatitis. Given its effects on ROS production and ability to modulate beta cell destruction due to autoimmunity in type 1 diabetes, haplogroup D may modulate the development of inflammation in the progression from simple steatosis to steatohepatitis.

The OR in our study for developing NAFLD with the *PNPLA3* rs738409 polymorphism is similar to previously published results.^47^ Our data suggest that an interaction exists between the *PNPLA3* rs738409 polymorphism and mitochondrial haplogroups, with haplogroup A potentially mitigating the pathogenic effects of rs738409. Numerous examples exist in the literature suggesting an interaction between the mitochondrial and nuclear genomes. Fluctuations in mitochondrial bioenergetic parameters can drive epigenetic changes via post‐translational modification of various proteins.^17^ This can change the expression of certain nuclear encoded bioenergetic genes. Mouse models have demonstrated by changing the mitochondrial DNA background of a particular strain that mtDNA can interact with nuclear DNA to affect cognition and can affect the inheritance of complex traits such as autoimmune encephalitis and anxiety‐related behaviours.^48^ A recent thorough characterization of the effects of mismatched nuclear and mitochondrial genomes revealed effects not only on mitochondrial function and ROS production but also changes in lipid metabolism, glucose and insulin homeostasis and ageing.^49^ Nuclear‐mitochondrial DNA exchange has also been shown to affect the development of liver steatosis in a mouse model.^14^ Variations in the mitochondrial genome have also been shown to affect the penetrance of nuclear genes, including *PNPLA3*.^10^

We therefore suspect that the pathogenicity of the *PNPLA3* rs738409 polymorphism may be diminished on the background of mitochondrial haplogroup A. Haplogroup A is defined by several polymorphisms including A4824G and C8794T.^40^ One study has showed that in Han Chinese patients showed that haplogroup A may decrease the risk of drug addiction.^50^ This study also showed that HeLa cells overexpressing 8794T had decreased levels of ROS production compared with those overexpressing 8794C.^50^ However, the exact mechanisms of how haplogroup A interacts with the *PNPLA3* rs738409 polymorphism and mitigates the risk of NAFLD remain unknown. It is also possible that haplogroup A and its related polymorphism may alter uncoupled respiration. It has recently been demonstrated that modulating adenine nucleotide translocase can increase uncoupled mitochondrial respiration leading to increased consumption of fatty acids and protection form NAFLD.^51^ It is possible that haplogroup A and its related polymorphisms may act in a similar fashion to affect coupling of respiration. Uncoupling mitochondrial respiration would be expected to lead to decreased mitochondrial ROS production.

Sex has an important impact on metabolism, and sex hormones affect mitochondrial metabolism. Females have a higher mitochondrial respiratory rate and lower oxidative stress, with both progesterone^52^, ^53^ and oestrogen^54^ playing important roles. Female mitochondria may be influenced by a higher expression of uncoupling proteins, which attenuate ROS production.^55^ Women have been shown to have a lower risk of NAFLD,^56^ potentially due to decreased fatty acid oxidation and increased lipogenesis in males.^57^ Consistently, we found that female sex was associated with decreased risk of NAFLD in multivariate regression models (Tables 3 and 4). Haplogroup G was associated with risk of NAFLD after controlling for sex and age >50 (as a surrogate for menopausal status) indicating that its mechanism of action is likely independent of the effect of sex on mitochondrial function and metabolism. Similarly, among patients with haplogroup A, the *PNPLA3* GG genotype was not associated with NAFLD regardless of sex (Table 4). This suggests that the ability of haplogroup A to mitigate the pathogenicity of *PNPLA3* GG is independent of sex. However, the small number of patients included in this study who were above 50 years of age makes it difficult to draw conclusions regarding the effect of menopausal status.

This study has several limitations. Our study only looked at patients who were diagnosed with NAFLD and not those with advanced stages of liver disease including hepatic steatohepatitis and cirrhosis. Therefore, we are only able to suggest which mtDNA variations may affect the initial development of NAFLD. Separate mtDNA changes may affect the development of steatohepatitis or cirrhosis. While viral aetiologies of hepatitis were ruled out based on serology, it is possible that other inherited causes of disordered lipid metabolism were not detected based on medical history alone. It is also possible that some of our control patients had early stages of hepatic steatosis, since ultrasound may not detect steatosis when <20% of hepatocytes contain fat droplets. Additionally, while we looked at sequence changes throughout the mitochondrial genome, we only looked at one nuclear change associated with NAFLD. Although, *PNPLA3* rs738409 is the most common variant associated with risk of developing NAFLD, other nuclear genes have been linked to hepatic steatosis. Therefore, a more thorough genome wide study looking at associations between the entire nuclear and mitochondrial genomes is necessary. However, this study would require a significantly larger number of patients to be adequately powered. Furthermore, although we can speculate as to the functional effects of mitochondrial haplogroups G and A in our patients, further mechanistic study will need to be carried out in order to assess their effects on mitochondrial function, including ETC enzymatic activity, membrane potential and coupling.

In conclusion, we demonstrate that mitochondrial haplogroup G is associated with an increased risk of NAFLD. We further demonstrate that patients with haplogroup A have a higher frequency of the *PNPLA3* rs738409 GG genotype without an increased risk of NAFLD. These results suggest a role for mtDNA in the development of NAFLD and that interactions between mtDNA and *PNPLA3* may modulate disease penetrance.

## CONFLICT OF INTEREST

AMG, YH, JC, CEM and SQ report no relevant conflicts of interest.

## AUTHOR CONTRIBUTIONS

AMG and YH designed and conducted the experiments, analysed data and wrote the manuscript. JC and CEM helped with experimental design and analysis and also edited the manuscript. SQ conceived the concept of the manuscript, oversaw all experiments and analysis and revised the manuscript. All authors have read and approved the final manuscript.

## Supporting information

Fig S1

Table S1‐S2

## Data Availability

Data are available upon request to the corresponding author.
