# Supplementary figures and images for: Mitochondrial haplogroup G is associated with nonalcoholic fatty liver disease, while haplogroup A mitigates the effects of PNPLA3
Source: Endocrinol Diabetes Metab. 2020 Oct 6;4(1):e00187. doi: 10.1002/edm2.187 (PMC7831202; doi:10.1002/edm2.187)

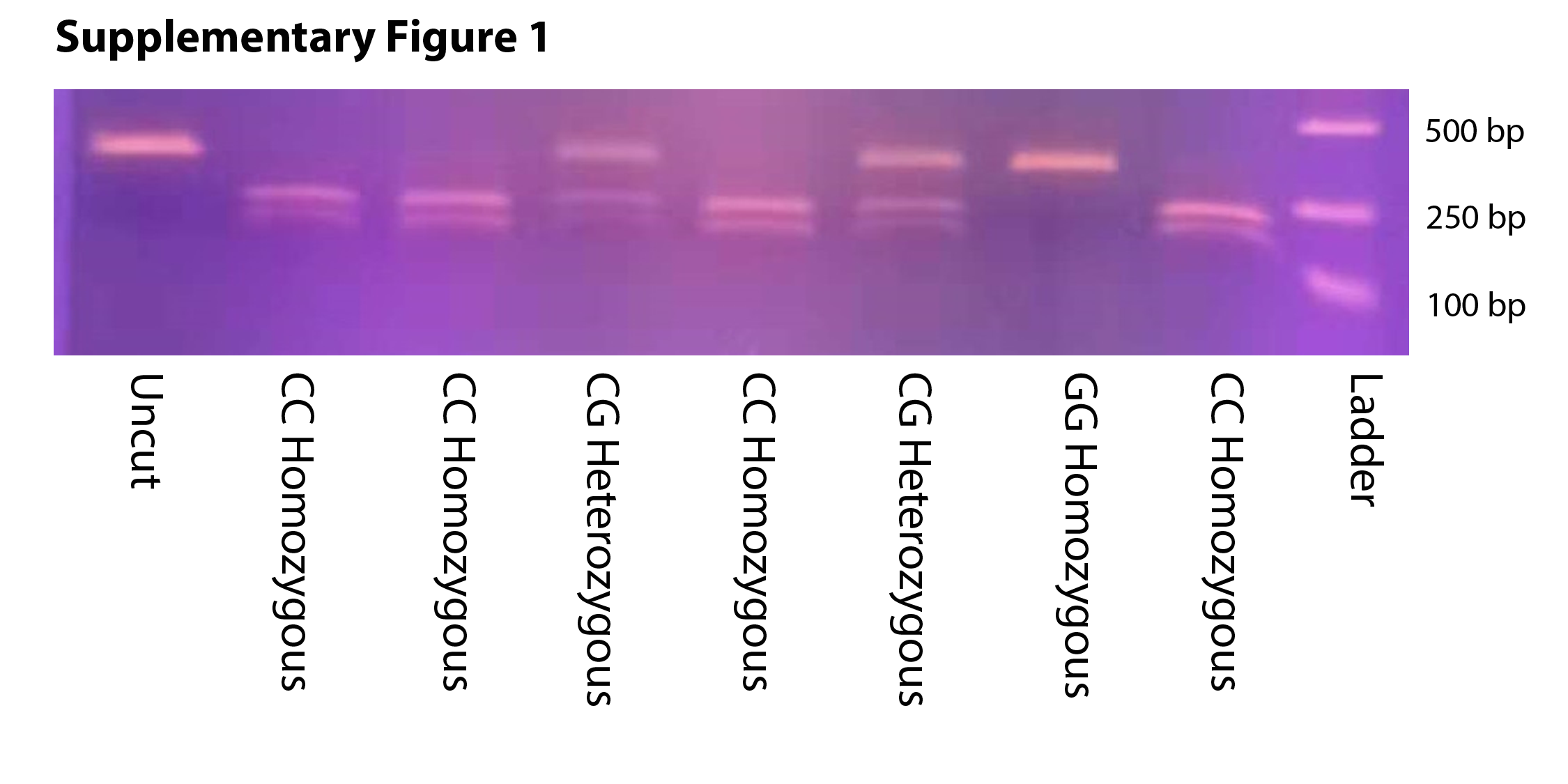

Supplement: Supplementary file 1 — Fig S1 [file EDM2-4-e00187-s001.tif]
