# Supplementary material for: Mitochondrial haplogroup G is associated with nonalcoholic fatty liver disease, while haplogroup A mitigates the effects of PNPLA3
Source: Endocrinol Diabetes Metab. 2020 Oct 6;4(1):e00187. doi: 10.1002/edm2.187 (PMC7831202; doi:10.1002/edm2.187)
Supplement: Supplementary file 2 — Table S1‐S2 [file EDM2-4-e00187-s002.docx]

**Supplemental Material**

Supplemental Figure 1: PNPLA3 genotyping. Results for a typical RFLP experiment are shown with each genotype. Examples of homozygous (CC), heterozygous (CG), and homozygous (GG) patients are shown.

Supplementary Table 1: Frequency of haplogroup D sub-haplogroups

|  | D4j | D4b | D4e | D5a | D5b | D5 | D4a | D4h | D4 | D4q | D4g | D1a | D4k | D6 | D1g | D1e | D6a | D4o |
| --- | --- | --- | --- | --- | --- | --- | --- | --- | --- | --- | --- | --- | --- | --- | --- | --- | --- | --- |
| Control | 6.8 | 17.1 | 4.3 | 12.8 | 6.8 | 7.7 | 8.5 | 4.2 | 11.1 | 0.9 | 4.3 | 0.9 | 1.7 | 1.7 | 0.9 | 4.3 | 0.0 | 0.0 |
| NAFLD | 6.2 | 20.2 | 6.2 | 12.4 | 10.8 | 4.7 | 11.6 | 2.3 | 7.8 | 0.0 | 3.1 | 0.0 | 0.8 | 0.0 | 0.0 | 5.4 | 0.8 | 0.8 |
| *P-*Value | 0.67 | 0.32 | 0.35 | 0.14 | 0.62 | 0.19 | 0.92 | 0.89 | 0.28 | 0.89 | 0.87 | 0.80 | 0.27 | 0.89 | 0.45 | 0.52 | 0.53 | 0.76 |

Supplementary Table 2: Frequency of each *PNPLA3* genotype stratified by mitochondrial haplogroup

|  | A | B | C | D | E | F | G | H | K | L | M | N | R | T | U | Y | Z | Total |
| --- | --- | --- | --- | --- | --- | --- | --- | --- | --- | --- | --- | --- | --- | --- | --- | --- | --- | --- |
| CC | 15  (4.3) | 50  (14.2) | 11  (3.1) | 78  (22.2) | 2  (0.6) | 46  (13.1) | 17  (4.8) | 23  (6.5) | 4  (1.1) | 2  (0.6) | 68  (19.3) | 14  (4.0) | 7  (2.0) | 1  (0.3) | 1  (0.3) | 2  (0.6) | 11  (3.1) | 352 |
| CG | 30  (6.3) | 47  (9.8) | 15  (3.1) | 121  (25.3) | 0  (0.0) | 67  (14.0) | 16  (3.3) | 34  (7.1) | 6  (1.3) | 1  (0.2) | 88  (18.4) | 16  (3.3) | 11  (2.3) | 2  (0.4) | 3  (0.6) | 7  (1.5) | 15  (3.1) | 479 |
| GG | 37  (11.3) | 46  (14.0) | 14  (4.3) | 71  (21.6) | 1  (0.3) | 39  (11.9) | 22  (6.7) | 11  (3.4) | 1  (0.3) | 1  (0.3) | 60  (18.3) | 11  (3.4) | 5  (1.5) | 0  (0.0) | 1  (0.3) | 1  (0.3) | 7  (2.1) | 328 |
| *P-Value* | ***0.001*** | 0.08 | 0.46 | 0.40 | 0.27 | 0.68 | 0.09 | 0.07 | 0.37 | 0.99 | 0.93 | 0.84 | 0.46 | 0.79 | 0.89 | 0.20 | 0.61 |  |
